# Design, Application, and Actionability of US Public Health Data Dashboards: Scoping Review

**DOI:** 10.2196/65283

**Published:** 2025-05-21

**Authors:** Gretchen Stahlman, Itzhak Yanovitzky, Miriam Kim

**Affiliations:** 1 School of Information Florida State University Tallahassee, FL United States; 2 School of Communication & Information Rutgers University New Brunswick, NJ United States

**Keywords:** dashboard, scoping review, public health, design, development, implementation, evaluation, user need, PRISMA

## Abstract

**Background:**

Data dashboards can be a powerful tool for ensuring access for public health decision makers to timely, relevant, and credible data. As their appeal and reach become ubiquitous, it is important to consider how they may be best integrated with public health data systems and the decision-making routines of users.

**Objective:**

This scoping review describes and analyzes the current state of knowledge regarding the design, application, and actionability of US national public health data dashboards to identify critical theoretical and empirical gaps in the literature and clarify definitions and operationalization of actionability as a critical property of dashboards.

**Methods:**

The review follows PRISMA-ScR (Preferred Reporting Items for Systematic Reviews and Meta-Analyses Extension for Scoping Reviews) guidelines. A search was conducted for refereed journal articles, conference proceedings, and reports that describe the design, implementation, or evaluation of US national public health dashboards published between 2000 and 2023, using a validated search query across relevant databases (CINAHL, PubMed, MEDLINE, and Web of Science) and gray literature sources. Of 2544 documents retrieved, 89 (3.5%) met all inclusion criteria. An iterative process of testing and improving intercoder reliability was implemented to extract data.

**Results:**

The dashboards reviewed (N=89) target a broad range of public health topics but are primarily designed for epidemiological surveillance and monitoring (n=51, 57% of dashboards) and probing health disparities and social determinants of health (n=27, 30%). Thus, they are limited in their potential to guide users’ policy and practice decisions. Nearly all dashboards are created, hosted, and funded by institutional entities, such as government agencies and universities, that hold influence over public health agendas and priorities. Intended users are primarily public health professionals (n=34, 38%), policy makers (n=30, 34%), and researchers or practitioners (n=28, 32%), but it is unclear whether the dashboards are tailored to users’ data capacities or needs, although 30% of articles reference user-centered design. Usability indicators commonly referenced include website analytics (n=22, 25%), expert evaluation (n=19, 21%), and users’ impact stories (n=14, 16%), but only 30% (n=26) of all articles report usability assessment. Usefulness is frequently inferred from presumed relevance to decision makers (n=17, 19%), anecdotal stakeholder feedback (n=16, 18%), and user engagement metrics (n=14, 16%) rather than via rigorous testing. Only 47% (n=42) of dashboards were still accessible or active at the time of review.

**Conclusions:**

The findings reveal fragmentation and a lack of scientific rigor in current knowledge regarding the design, implementation, and utility of public health dashboards. Coherent theoretical accounts and direct empirical tests that link usability, usefulness, and use of these tools to users’ decisions and actions are critically missing. A more complete explication and operationalization of actionability in this context has significant potential to fill this gap and advance future scholarship and practice.

## Introduction

### Background

The disjointed public health response to the COVID-19 pandemic in the United States highlighted the critical importance of having robust public health data systems in place and the potential utility of data dashboards for ensuring timely and unrestricted access to critical public health data [1,2]. The ubiquitous and prominent use of dashboards to chronicle the progression and public health response to the COVID-19 pandemic has increased the appeal of these tools to a broad and diverse range of decision makers, including public health leaders and professionals, health care providers, community leaders, policy makers, and advocates [3,4]. Data dashboards are frequently touted as cost-effective means to share and access public health and other types of publicly available data because they transform complex data into intuitive information displays, afford instantaneous and near-universal access of multiple stakeholders to data-based insights, and allow users to explore data on their own to answer questions that are important to them [5-8]. They are also increasingly recognized for their democratizing potential, both in terms of making data available to a wider and more diverse range of audiences and ensuring that diverse stakeholders, particularly those who are less privileged and are most likely to be impacted by how data are interpreted and used in decision-making, have the power and opportunity to shape what and how data are used in this context [9].

### Aims and Contributions

As public health data dashboards are poised to become more integral to public health decision-making at the local, state, and federal levels in the United States, it is imperative to proactively consider how they may be best designed, implemented, improved, and sustained to promote sound, equitable, and effective public health policies and practices [3,10]. Progress in this direction is currently impeded by the fragmented nature of research on this topic, specifically the lack of coherence regarding effective dashboard design principles and practices, as well as the mechanisms, factors, and supports that make dashboards usable and useful to diverse user groups and across health and decision-making contexts [3,7,10,11]. Previous reviews of the literature on the use of data dashboards in public health have generally focused on identifying and assessing the utility of key design features of dashboards but were limited to specific public health applications, such as COVID-19 [2,12], food and nutrition systems [13], infectious diseases [14], and environmental hazards [15], or were limited in focus to specific dashboard design features, such as data visualizations [16] or usability [4]. Thus, a systematic review of the literature that is broader and more comprehensive in the scope of health topics and applications considered and that goes beyond design-related research questions to consider different goals of data dashboards (eg, alert, educate, and persuade), theories of action (or how dashboards are presumed or expected to work), and outcomes of use (including impact indicators) has significant potential to advance the scientific study of data dashboards as instruments for promoting sound health-related decisions, policies, and practices. Accordingly, the primary objective of this scoping review is to describe and critically assess the current state of scientific knowledge regarding the design, application, and actionability of US national public health data dashboards; note critical theoretical and empirical gaps; and identify potential venues for improving knowledge integration.

An additional unique contribution of this scoping review is the explicit focus on *actionability* as a critical feature of effective public health data dashboards. There has been a growing interest in the question of what makes public health data dashboards *actionable*, that is, ensuring they provide an optimal match for both purpose and use [17-21]. However, the concept of actionability in the context of public health data dashboards remains poorly defined and insufficiently developed to effectively guide their design and implementation. Ivanković et al [22], for example, defined data dashboard actionability according to seven features: (1) knowing and clearly stating the desired consumers of the information, (2) selection and presentation of appropriate indicators, (3) clearly stating the sources of data and methods used to generate indicators, (4) demonstrating variation over time and linking changes to public health interventions, (5) providing as high a spatial resolution as possible to enable consumers to evaluate local risk, (6) disaggregating data to population subgroups to further enable evaluation of risk, and (7) providing narrative information to enhance interpretation of the data by the consumer. This *functional conception* understands actionability as a function of both usability and degree of match between data and users’ information needs, which is intuitive but may not be equally applicable across audiences and settings [20]. Other scholars in this space offer a *behavior-centered conception* of actionability [21]. In their view, to be actionable, dashboards must prompt or trigger users to act on data by being integrated, via behavioral design, into users’ data use practices or routines, such as assessing performance on tasks or progress on goals. Finally, there are those who advocate for a *decision-centered conception* of actionability, whereby data dashboards are considered actionable to the extent that they provide data, analyses, and forecasts (eg, predictive analytics), allowing decision makers to make an informed choice among alternatives [19,20,23]. Accordingly, an additional important objective of the scoping review is to extract, reconcile, and integrate different conceptions and operationalizations of actionability across studies for the purpose of advancing a more complete explication and a standard approach to the measurement of actionability as a critical design element of public health data dashboards.

## Methods

### Review Methodology and Protocol

This scoping review was designed to generate both descriptive and thematic accounts of the purpose; intended audiences; range of health topics; design elements and characteristics; usability and usefulness measures; theories of action; and logistics of developing, implementing, and sustaining public health data dashboards based on information available from published US case studies. Given the considerable diversity in research questions and methodologies used across disciplines and fields to study public health data dashboards, a scoping review of the literature is most appropriate for producing a systematic evidence synthesis [24]. This study followed the PRISMA-ScR (Preferred Reporting Items for Systematic Reviews and Meta-Analyses Extension for Scoping Reviews), which is the most up-to-date and advanced approach for conducting and reporting scoping reviews [25]. In the subsequent sections, we briefly describe the methodological processes implemented. Further details are available in the published protocol [26].

### Selection Criteria, Sources, and Search Strategy

For the purposes of this scoping review, we defined *public health data dashboard* as a publicly accessible, web-based, interactive, and regularly updated information management and data visualization tool that displays and tracks population health indicators, metrics, and data points. This definition is inclusive of a broad range of population health–relevant data, such as epidemiological surveillance, but excludes the use of data dashboards in clinical and health care organizations as well as dashboards incorporated into patient portals. Textbox 1 displays all other inclusion and exclusion criteria used for searching and retrieving relevant publications. Given the rapid advancements in dashboard technology in recent years, adopting a broader historical perspective dating back to the beginning of the century can be useful for determining what, if anything, changed over time regarding the design philosophies and theories of action guiding the development and implementation of these tools. To ensure adequate and inclusive representation of empirical studies, no methodological orientation restrictions were imposed as selection criteria.

**Publications inclusion and exclusion criteria.**

**Inclusion criteria**

- Publication type: Full text, peer-reviewed journal articles, conference proceedings, book chapters, or published reports
- Language: English
- Scope and focus: Empirical (qualitative, quantitative, or mixed methods) case studies of design, implementation, and evaluation of a US-located national public health dashboard
- Publication date: 2000 to 2023

**Exclusion criteria**

- Publication type: Peer-reviewed abstract-only or publications for which full text is not available; non–peer-reviewed publications
- Language: Non-English
- Scope and focus: Commentaries, background papers, or reviews of literature; case studies of dashboards located outside of the United States; case studies of state public health dashboards; or case studies of dashboards in clinical or health care settings
- Publication date: Before 2000 or after 2023

The search methodology (refer to the published protocol for full details [26]) involved a series of steps to minimize potential errors in our search strategies that negatively affect the quality and validity of this scoping review [27]. First, in collaboration with a research librarian, we searched both the Medical Subject Headings (MeSH) database and keywords listed in recently (2019 and onward) published journal papers on the topic of public health data dashboards to identify the most relevant keywords and terms for searching for relevant publications that meet our inclusion criteria. In the next step, we followed an established procedure [28] to experiment with different combinations of databases and search queries to optimize the recall (sensitivity) and precision (specificity) of our search strategy. Given the aims of this scoping review, we opted for a search strategy that maximizes coverage, that is, will increase the likelihood of identifying all or as many relevant publications as possible. Accordingly, we searched CINAHL, PubMed, MEDLINE, and Web of Science databases in June 2023 for published research reports using the least restrictive validated search query ([“dashboard” OR “data dashboard” OR “information visualization” OR “data visualization”] AND [“public health” OR “population health”]). These databases were selected because they were identified, via rigorous testing, as providing optimal coverage of research published across a broad range of disciplines and fields [29]. We conducted supplementary searches of gray literature using the same search query to search OpenGrey for additional documents that met all selection criteria.

### Data Charting

The list of themes and variables used for data abstraction is presented in Textbox 2. This list was created following an iterative process of reviewing the strategies and instruments used in previous similar reviews; consultations with an expert advisory group composed of public health data dashboard creators; and pretesting of the instrument with a randomly drawn sample of publications included in the review using the same procedure described in the Selection Criteria, Sources, and Search Strategy section for validating the screening and selection procedure, including training on the task and tests of intercoder agreement (refer to the published protocol for full details [26]).

Data were extracted and recorded using a survey instrument designed to capture a range of closed-ended, multiple, and open-ended responses to facilitate standardized coding by multiple coders. Quantitative data were cleaned, harmonized, and properly labeled before being analyzed using SPSS Statistics (version 29; IBM Corp) for generating descriptive statistics. Open-ended text entries were reviewed and analyzed collectively by the authors and organized into common themes to produce additional insights.

**List of data extraction elements.**

**Study identifiers**

- Metadata (title, authors, journal, year of publication, and keywords)
- Study type (eg, descriptive, exploratory, and explanatory)
- Research methodology
- Study focus (eg, development, implementation, and evaluation)
- Geographic location (country)

**Data characteristics**

- Data sources
- Health topics
- Type of data (eg, epidemiological, health services, and behavioral)
- Populations represented in the data
- Indicators or metrics selected for visualizations
- Data level of granularity (eg, national, state, county, and city)

**Dashboard design characteristics**

- Stated goals or purposes of the dashboard (eg, tracking or monitoring)
- Design philosophy cited (eg, user-friendly, functional, and co-design)
- Design process (eg, iterative and collaborative)
- Dashboard features (eg, customization and search functionalities)
- Data visualization tools (eg, maps, graphs, and tables)

**Users and usability**

- Intended audiences
- Public access (open, restricted or limited, and requires registration)
- Dissemination channels (eg, social media, news outlets, email, and listserv)
- Reported use- or usability-related barriers or challenges

**Logistics or operation**

- Ownership or hosting
- Source of funding
- Software tools (commercial and open source)
- Data updating and quality assurance protocols
- Technical support (eg, user manuals, training, and customer service option)

**Performance and usefulness or impact evaluation**

- Evaluation methodology
- Use or usability indicators captured (eg, website analytics and user ratings)
- Impact indicators or other evidence of impact
- Explanations given for observed effects or impact (or lack of)

## Results

### Overview

**Figure 1:**
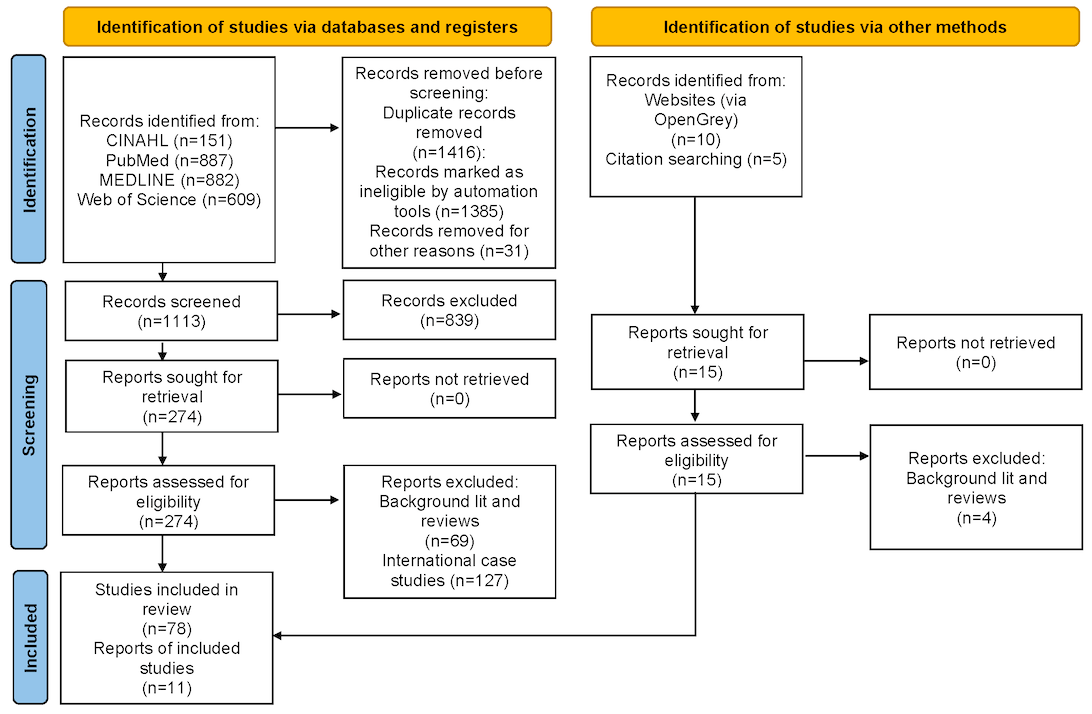
PRISMA flowchart.

A total of 2529 documents (peer-reviewed journal papers, conference proceedings, and book chapters) were initially retrieved by implementing the search procedure. After the removal of duplicate results (1386/2544, 54.48%) and the addition of “grey literature” sources (10/2544, 0.39%) and additional papers identified through snowballing of sources cited in other related literature reviews (5/2544, 0.2%), 44.34% (1128/2544) of documents were retained for manual screening and 8.49% (216/2544) met the study’s definition of a case study of a public health dashboard. Of these 216 documents, 127 (58.8%) were excluded because they were case studies of public health data dashboards in countries outside the United States and therefore beyond the scope of this scoping review. However, these items were retained for the purpose of conducting a future complementary scoping review to compare findings across international boundaries. Accordingly, 89 US-based case studies of public health data dashboards that met all selection criteria were included in the scoping review. The reasons for exclusion are detailed in the PRISMA (Preferred Reporting Items for Systematic Reviews and Meta-Analyses) diagram (Figure 1), and the PRISMA-ScR reporting checklist is presented in Multimedia Appendix 1.

Each published case study of a US-based public health data dashboard was coded using the data charting instrument (Textbox 1). All coders (n=5) first received training on the task and then were provided with a random sample of 10 documents to code. Agreement among coders was assessed using Krippendorff α [29], and the omnibus test result was significantly lower (α=.37) than the acceptable standard (α=.70). Coders then received additional training on the task and then independently coded a fresh set of 10 randomly selected documents. Intercoder agreement was reassessed and reached an acceptable standard (α=.78), with any ambiguities regarding coding resolved via a full team review and consensus.

### Study Characteristics

A list and basic characteristics of the case studies included in the review are provided in Multimedia Appendix 2 [8,30-117]. Articles reviewed were published in 60 different outlets between 2004 and 2023 and most commonly appeared in the American *Journal of Public Health* (7/89, 8%), the *Journal of the American Medical Informatics Association* (6/89, 7%), the *Journal of Public Health Management and Practice* (5/89, 6%), and *JMIR Public Health and Surveillance* (3/89, 3%). While the case studies included in this scoping review were published over a period of 19 years (2004-2023) and are quite diverse in terms of health topics and intended users of dashboards, a majority (61/89, 69%) were published after 2019, coinciding with the COVID-19 pandemic. Indeed, 40% (35/89) of the case studies included in the review directly address some aspect of COVID-19 and public health.

There was a considerable variation in the type of studies included in the scoping review. Over half (49/89, 55%) provided a description of the dashboard developed, including sources of data, design features, and technical details. About a quarter (23/89, 26%) were more exploratory in nature, reporting the results of usability tests conducted with users and any subsequent refinement of the dashboard developed. A smaller number of case studies (13/89, 15%) were classified as explanatory, as they included qualitative or quantitative assessment of the degree to which use of the dashboard was associated with effects on users’ knowledge, decisions, or actions. A handful of cumulative case studies (4/89, 5%) considered lessons learned from comparing the development or implementation of a dashboard across settings or user groups. Regarding case study methodology, 10% (9/89) of the case studies included in the review used quantitative methods, 37% (33/89) used qualitative methods, and 31% (28/89) combined mixed methods. About 20% (18/89) of the case studies reviewed were a description of a dashboard and the process of developing the dashboard.

Overall, case studies that systematically assess use, usefulness, and outcomes of using public health dashboards remain scarce even as the volume of published empirical research on the topic has sharply risen in recent years. This is also evidenced in the types of information frequently provided in the case studies reviewed. Information typically reported includes features or functionalities of the dashboard (80/89, 91%), sources of data used (78/89, 89%), and the logistics of developing and deploying the dashboard (62/89, 71%). Less frequently reported is information pertaining to assessing use or usability of the dashboard (26/89, 30%), results of usability tests (19/89, 22%), any form of impact evaluation (17/89, 19%), or dissemination procedures (14/89, 16%). This distribution may reflect authors’ decisions about what information to report due to space constraints and the absence of standards for reporting on dashboards, but it may also point to the paucity of efforts to assess the usefulness of these tools for public health decision makers.

### Dashboard Hosting and Funding Source

As shown in Table 1, the dashboards represented in the case studies included in the scoping review were most likely to be hosted on university websites, compared to federal government sites, sites maintained by nonprofit or philanthropic organizations, state government sites, and independent hosts. A handful of dashboards (3/89, 3%) were hosted by health care industry organizations. Website hosting information was unavailable for a third of the case studies reviewed, primarily because a web address for the dashboard was not provided.

The findings summarized in Table 1 also demonstrate that most of the dashboards studied were funded by US government health agencies (eg, Centers for Disease Control and Prevention [CDC], National Institutes of Health, and Agency for Healthcare Research and Quality), followed by universities and foundations, with grants being the most common mechanism for funding the development and deployment of public health dashboards (39/89, 44%). Funding information was not provided for a third of the case studies (30/89, 34%) reviewed. However, for case studies where funding information was provided, 53% (19/36) of federally funded studies used federal data sources, compared to other data sources, such as state agencies (13/36, 36%), research organizations (12/36, 33%), media sources (11/36, 31%), and local agencies (6/36, 17%). Taken together, these findings suggest that institutional actors, such as government agencies, universities, and philanthropic organizations, are the primary funders, developers, and hosts of public health data dashboards in the United States, presumably because they possess the necessary resources and expertise to create and maintain these tools. However, this may be a source of potential selection bias regarding topics, data, and indicators covered by these dashboards.

**Table 1:** Dashboard hosts and sources of funding (N=89).

		Value, n (%)
**Dashboard hosting**		
	University websites	23 (26)
	Federal government websites	9 (10)
	Health management organizations	9 (10)
	Nonprofit and philanthropic organizations	7 (8)
	State government websites	6 (7)
	Independent and nonaffiliated websites	6 (7)
	Unknown	29 (33)
**Source of funding**		
	Federal health agencies	36 (41)
	Universities	19 (22)
	Foundations	13 (15)
	Unknown	23 (53)

### Topic, Purpose, and Intended Users

A complete list of public health topics covered by the dashboards represented in this review is included in Multimedia Appendix 2. For the purpose of this analysis, case studies of public health dashboards were grouped according to type of data used and purpose of presenting the data such that they map onto key public health functions. As shown in Table 2, the primary function or purpose of the dashboards reviewed was surveillance and monitoring. Epidemiological surveillance was the most common purpose of the data presented in dashboards, followed by health outcomes surveillance (eg, births, deaths, life expectancy, and quality of life measures), tracking of use of health services (eg, proportion of population screened or immunized), and analysis of sources or causes of health disparities (eg, social determinants of health). Behavioral surveillance such as tracking self-reported attitudes and behaviors, news and social media content monitoring, health policy or legislation tracking, and tracking availability of health care facilities or health services in a certain geographical area were less common by comparison. These differences may be attributed to the limits imposed by the types of population-level health data available to dashboard developers, which are predominantly of the epidemiological and health services type. Notably, about 22% (20/89) of the dashboards reviewed were equipped to provide predictions of future trends (often based on data extrapolation) or likely effects (positive or adverse) of policies, such as increasing access to health care insurance or services in a community, which may enhance their actionability.

Recognizing that public health data dashboards are often created to serve multiple audiences, the intended users of dashboards identified by the authors of the case studies reviewed, as shown in Table 2, were most commonly public health decision makers (eg, public health departments and officials), followed by policy makers (eg, agency, state, and city administrators), researchers (eg, researchers, analysts, and academics), practitioners (eg, clinicians, health care administrators, public health professionals, and first responders), and the general public. By comparison, public health advocates were the least likely to be identified as potential intended users of dashboards. Intended users of the dashboard were not explicitly identified in 17% (15/89) of all case studies did not explicitly identify intended users of the dashboard.

Because dashboard actionability is primarily a function of the match between audience needs and the purpose of presenting data (data affordances), a multiple-response cross-tabulation analysis was conducted to probe the degree to which data affordances of dashboards are tailored to various users. As shown in Table 3, the results of this analysis demonstrate no clear pattern of covariation of dashboards’ data affordances by groups of intended users. Thus, case studies of dashboards designed for epidemiological surveillance were equally likely to identify researchers, policy makers, and public health decision makers or practitioners as intended users, and the same was true for dashboards designed for tracking and comparing health outcomes and those designed to highlight the effects of social determinants of health. By comparison, case studies of dashboards designed for tracking access and use of health services were more likely to identify members of the general public as the intended audience compared to researchers, policy makers, and public health decision makers. This inconclusive pattern of association suggests that the design of actionable dashboards tailored to specific audience groups is not a common practice. Conversely, it may reflect dashboard designers’ belief that the dashboards they design are universally usable and useful for diverse audience groups and for diverse purposes.

**Table 2:** Primary focus and intended audiences of dashboards (N=89)^a^.

		Value, n (%)
**Dashboard focus**		
	Epidemiological surveillance	51 (57)
	Health outcomes surveillance	34 (38)
	Use of health services	29 (33)
	Health disparities	27 (30)
	Behavioral surveillance	13 (15)
	News and social media surveillance	11 (13)
	Policy or legislative surveillance	9 (10)
	Services availability	15 (17)
**Intended audiences**		
	Public health decision makers	34 (38)
	Policy makers	30 (34)
	Researchers	28 (32)
	Practitioners	28 (32)
	General public	27 (30)
	Advocates	12 (14)
	None explicitly referenced	15 (17)

^a^As dashboards frequently incorporate different types of data that serve multiple functions and cater to multiple user groups, the total percentage across categories exceeds 100%.

**Table 3:** Relationship between the purpose of presenting data and intended users of dashboards (N=89)^a^.

	Researchers, n (%)	Policy makers, n (%)	Decision makers, n (%)	Practitioners, n (%)	Advocates, n (%)	General public, n (%)
Epidemiological surveillance	16 (57)	18 (60)	21 (62)	14 (50)	5 (42)	21 (78)
Behavioral surveillance	3 (11)	8 (27)	6 (18)	6 (21)	2 (17)	3 (11)
Policy surveillance	2 (7)	4 (13)	3 (9)	4 (14)	1 (8)	2 (7)
News and social media surveillance	3 (11)	5 (17)	5 (15)	4 (14)	2 (17)	5 (19)
Access to services monitoring	3 (11)	7 (23)	5 (15)	4 (14)	3 (25)	9 (33)
Use of services monitoring	5 (18)	7 (23)	10 (29)	8 (29)	4 (33)	9 (33)
Health outcomes surveillance	11 (39)	13 (43)	13 (38)	10 (36)	6 (50)	12 (44)
Health disparities	10 (36)	10 (33)	14 (41)	11 (39)	5 (42)	9 (33)
Prediction	7 (25)	5 (17)	5 (15)	2 (7)	1 (8)	4 (15)

^a^Percentages indicate the distribution within individual subsets rather than across all cases.

### Data Source, Focus, and Representation

Table 4 describes the distribution of sources and types of data of the dashboards included in the review. About half of the dashboards (44/89, 49%) used data from federal agency sources (eg, CDC and Agency for Healthcare Research and Quality), and a third (29/89, 33%) used data collected by state agencies (eg, state department of health). Data obtained from health care facilities (eg, administrative data such as emergency room records and hospitalizations) were used by a quarter of all dashboards (23/89, 26%), and media data and data collected by research organizations such as universities were each a data source used by a fifth of all dashboards (19/89, 21%). Aggregated patient or clinical data (14/89, 16%), municipal data (13/89, 15%), and insurance claims data (7/89, 8%) were less frequently used by the dashboards included in the review.

The type of data featured in dashboards was primarily epidemiological data (eg, incidence of disease, illness, or events such as drug overdoses), health services data (eg, data about services provided by certified health providers, such as hospitalization, ambulatory care, screens, medications, and immunizations), clinical data (eg, data related to patient diagnosis, exposures, and laboratory tests), and health outcomes data (eg, births, deaths, life expectancy, and quality of life indicators). Behavioral data (eg, self-reported measures of beliefs, attitudes, and behaviors), media data (eg, news coverage of health topics or social media posts), and environmental risk data were less frequently integrated into the dashboards reviewed, presumably because such data are not routinely collected or readily available to creators of public health data dashboards.

The same pattern of findings emerged regarding the range of public health issues addressed by the dashboards studied (refer to Multimedia Appendix 2 for the complete list): common categories of issues included risk factors (eg, chemical exposure, infectious diseases, and tobacco use; 42/89, 47%); disease incidence (eg, obesity and diabetes; 22/89, 25%); health disparities (eg, access or use of health and medical services; 17/89, 19%); and, less frequently, social determinants of health (5/89, 6%) or behavioral or public opinion insights (5/89, 6%). Most dashboards (81/89, 91%) focused on a single topic, with about 40% (35/89) of the dashboards studied exclusively focused on the topic of COVID-19.

Regarding representation, the dashboards studied afforded users access to varying levels of international (15/89, 17%), national (41/89, 46%), state (47/89, 53%), and hyperlocal (eg, city or town and county; 62/89, 70%) public health data, with the greatest degree of overlap between state and local data (37/89, 42%). There were also notable variations in the populations represented in the data used by dashboards. Patient populations (48/89, 54%) and the general population (46/89, 52%) were most frequently represented in the data used compared to provider (health care professionals and medical institutions and organizations) populations (7/89, 8%) and data that exclusively represents populations considered vulnerable (10/89, 11%). As may be expected, populations considered vulnerable were more likely to be represented in dashboards focused on health disparities and social determinants of health (6/10, 60%) than dashboards focused on other aspects or dimensions of public health (eg, risk factors and use of health services).

**Table 4:** Source and type of data included in dashboards (N=89)^a^.

			Value, n (%)
**Data source**			
	Federal agency	44 (49)	
	State agency	29 (33)	
	Health care facilities	23 (26)	
	Media	19 (21)	
	Research organizations	18 (20)	
	Aggregated patient data	14 (16)	
	Municipal data	13 (15)	
	Insurance claims data	7 (8)	
**Data type**			
	Epidemiological data	48 (54)	
	Health services data	48 (54)	
	Clinical data	41 (46)	
	Health outcomes data	38 (43)	
	Behavioral data	19 (21)	
	Media data	5 (6)	
	Environmental risk data	4 (5)	

^a^As dashboards used data from multiple sources and of different types, total percentage across categories exceeds 100%.

### Dashboard Design Process and Design Principles

On the basis of the information provided by authors, we determined that the design of dashboards included in the review was most frequently driven by the intended purpose or goal of using the dashboard (*functional design*, represented in 29/89, 33% of case studies) or the needs or preferences of users (*user-centered design*, represented in 28/89, 32% of case studies), but less frequently to facilitate or support a particular decision-making process (*decision-centered design*, represented in 13/89, 15% of case studies). No clear design philosophy was discussed by authors in 35% (31/89) of the analyzed case studies.

Variations regarding the collaborative nature, if any, of the design process ranged from creator-driven (no input from users, represented in 39/89, 44% of case studies), through creator-driven with user feedback (27/89, 30% of case studies), and to a partnership-based or co-design process (23/89, 26% of case studies). Reported collaborations on dashboard development were overwhelmingly scientific collaborations with external experts or teams of developers (45/89, 51%) and less likely to involve collaborations with funders (10/89, 11%), community representatives (10/89, 11%), or industry (6/89, 7%). Over one-third of all case studies (32/89, 36%) did not reference any collaboration.

Given that relatively few case studies involved user feedback or collaboration, data visualization choices were presumably made without input from users in many cases. Nevertheless, data visualization tools referenced include graphs and charts (69/89, 78%), maps (54/89, 61%), timelines (36/89, 40%), and tables (32/89, 36%), with information about visualization tools missing from 8% (7/89) of case studies analyzed. Interactive customization options referenced include selecting or sorting cases by ≥1 indicators (54/89, 61%), selecting or grouping cases by location (46/89, 52%), sorting or grouping by time (34/89, 38%), sorting or grouping by demographic characteristics (25/89, 28%), and a searching function (10/89, 11%). Information regarding customization was missing or ambiguous in 23% (20/89) of case studies. In 30% (27/89) of case studies, authors indicated that integrating health data with social determinants of health data for the same group or locality (eg, rural health indicators by rural access to broadband internet) was possible.

Data visualizations implemented in the dashboards studied could be most frequently disaggregated spatially or geographically (52/89, 58%), followed by temporally or time (eg, year and month; 32/89, 36%). Other common disaggregation options reported include demographics (eg, age, gender, race, and ethnicity; 24/89, 27%) and socioeconomic factors (eg, education and income; 21/89, 24%). No disaggregation options were referenced in 23% (20/89) of case studies analyzed, and disaggregation by contextual factors (eg, environmental hazards (11/89, 12%), health services availability (7/89, 8%), and genomic and biological factors (3/89, 3%) was available in <15% of case studies analyzed. Interestingly, we found no reference to data storytelling, simulations, and other more interactive forms of audience engagement with data in the case studies reviewed.

### Use, Usability, and Usefulness

To determine whether a dashboard was still active at the time of conducting our review, we used the URL provided by authors, either in the text of the publication or in any supporting materials. URLs of dashboards were not provided in 41% (36/89) of the case studies analyzed. We were able to confirm that 47% (42/89) of all dashboards were still active at the time of our review and that 12% (11/89) were inactive or could no longer be accessed due to broken links. This seems to be influenced more by relevance or data availability than by the time elapsed since the case study was published. Thus, while recent, 45% (16/35) of the case studies of COVID-19 dashboards were no longer available or accessible at the time of producing this scoping review, and many of those that remain accessible have not been recently updated, as COVID-19 cases and death data reporting has been discontinued by CDC with the end of the public health emergency in May 2023. In addition, based on information provided in the case study or by inspecting the URLs provided, we were able to determine whether users had unrestricted or conditional access to dashboards included in the analysis. Open or unrestricted public access to the dashboard was observed in 46% (41/89) of all cases, whereas conditional access (eg, having to register as a user before being granted access) was observed for 14% (12/89) of all cases.

Only sparse information was provided in the case studies reviewed regarding how users were to learn about the availability and intended use of data dashboards, with such information not reported for 75% (67/89) of cases. Dissemination channels referenced when such information was provided include webinars, training, and outreach (13/89, 15%); social media posts (5/89, 6%); newsletters (4/89, 5%); news items, email distribution lists, and blogs (3/89, 3% each); and targeted advertising or website information (2/89, 2% each).

Usability indicators referenced include website analytics (22/89, 25%); experts’ evaluation (19/89, 21%); users’ impact stories (14/89, 16%); user ratings (12/89, 14%); citations, references, and mentions (8/89, 9%); and URL links (eg, external sites that link to or embed dashboards; 2/89, 2%). Usability information was not provided for 47% (42/89) of case studies included in the review. Indicators of usefulness (eg, impact on user knowledge, perceptions, decisions, or actions) mentioned in case studies include expectations regarding public health impact (17/89, 19%), stakeholder feedback or use (16/89, 18%), user engagement metrics (14/89, 16%), citations or references to dashboards in academic publications (12/89, 14%), and anecdotal evidence of association between policy makers’ use of dashboards and policy actions (6/89, 7%). Information about impact indicators was not provided for 51% (45/89) of the case studies reviewed.

### Actionability Assessment

In addition to producing an updated, state-of-the-art review and analysis of public health data dashboards in the United States, a primary motivation for conducting this scoping review was to clarify the meaning and significance of *actionability* as a property of effective public health data dashboards. Our findings distinguish among 3 principal conceptions of dashboard actionability. A common conception, popularized by Ivanković et al [22], understands actionability as the degree of match between purpose and use and associates it with functional design such that an actionable dashboard displays information clearly and efficiently, is intuitive to use, and is easily customizable to allow data exploration. Our analysis revealed that 33% (29/89) of the case studies of dashboards reviewed used functional design. Table 5 assesses the applicability of the actionability criteria proposed by Ivanković et al [22] to the case studies included in the review at the aggregate, recognizing that this scheme was developed to assess actual dashboards (as opposed to research reports on dashboards).

Several valuable insights emerge from this exercise. First, dashboard actionability critically depends on the availability of the “right data”—not simply in terms of quality, relevance, and timeliness but also the degree of data granularity and adequate representation of both subpopulations and relevant indicators. The “right data” also has much to do with public health focus: most case studies of dashboards reviewed were designed for epidemiological or health services access or use surveillance; only a handful were intentionally designed to support other critical public health missions, such as health education and prevention, health policy advocacy, and improved access to health services. Thus, expanding the types and diversity of data incorporated into dashboards is necessary for enhancing the actionability of these tools. Second, actionability is also a function of match to purpose and use, which varies depending on the goal of data use (eg, surveillance vs analysis or prediction) and the range of questions that can be answered given the data layering and customization possibilities afforded by a dashboard. This dimension of actionability is acutely relevant for exploring or analyzing data in context: <15% (5/89) of case studies of dashboards included in the review afforded users the opportunity to explore the relevance or significance of contextual factors such as social determinants of health. Third, the use of dashboards can result in unintended or undesirable effects [3]. This may be due to bias in the data used for creating a dashboard [118], bias associated with the presentation of data [119], or bias (whether intentional or unintentional) that affects the correct interpretation or proper use of insights drawn from data. Therefore, actionability requires acknowledgment of any actual and potential limitations or sources of bias that may influence dashboard use. This necessarily means going beyond mere transparency regarding data sources, methods, and funding to introducing, as a matter of standard practice, built-in guardrails against uninformed or improper use of dashboards in the form of alerts or cautions, disclaimers, and perhaps even recommendations or guidelines regarding acceptable use.

**Table 5:** Applicability of actionability criteria.

Actionability criterion	Scoping review findings
Knowing and clearly stating the desired consumers of the information	Information about intended users was available for most of the case studies reviewed (74/89, 83%). The primary intended audiences identified were public health decision makers, policy makers, and researchers, with secondary audiences including practitioners, advocates, journalists, and the general public.
Selection and presentation of appropriate indicators	Virtually all case studies reviewed used indicators that were topic relevant and aligned with the stated purpose of the dashboard. However, in only about half of all cases (50/89, 56%) appropriate indicators were determined after consulting intended users of a dashboard. Choice of indicators appears to be constrained by data availability.
Clearly stating the sources of data and methods used to generate indicators	Sources and types of data were clearly noted in most case studies reviewed. However, there was less transparency regarding methods (not reported in 31/89, 35% of case studies), software used (not reported in 25/89, 28% of cases), and collaborators, if any (not reported in 32/89, 36% of cases). There was no reference in the case studies reviewed to the inclusion of disclaimers regarding data limitations, although it is possible that disclaimers were included in some or most cases.
Demonstrating variation over time and linking changes to public health interventions	About 40% (36/89) of case studies reviewed included visualization of visualization of indicators, and 34% (34/89) allowed for temporal customization of data. None were linked to the effect of a public health intervention, although about 22% (20/89) of cases involved dashboards capable of extrapolating predictions. Still, this criterion does not universally apply to all dashboards, given variations in purpose and type of data used.
Providing as high a spatial resolution as possible to enable consumers to evaluate local risk	Most dashboards represented in the case studies reviewed (62/89, 70%) had a degree of data granularity extending to the local level. Still, this criterion does not universally apply to all dashboards, given variations in purpose and the scope and quality of local data available.
Disaggregating data to population subgroups to further enable evaluation of risk	About 27% (24/89) of the case studies of dashboards reviewed allowed for data disaggregation by demographics, 24% (21/89) for disaggregation by socioeconomics, and 8% (7/89) for disaggregation based on health insurance status. However, this particular affordance of public health dashboards is likely more common. At the same time, only 11% (10/89) of the cases of dashboards reviewed used data specific to a particular subgroup, which may indicate inadequate representation of minoritized groups and other groups considered vulnerable that are underrepresented in general population data.
Providing narrative information to enhance interpretation of the data by the consumer	The findings of the scoping review do not reveal a standard approach to the inclusion of narrative information to aid interpretation. Such information was rarely included in the case studies reviewed, and most (67/89, 75%) did not include any information pertaining to dissemination to users.

A second and equally common conception of actionability emerging from the case studies of dashboards reviewed (28/89, 32%) is behavioral or user-centered design. This conception primarily understands dashboard actionability as a function of both usability and usefulness: dashboards can support evidence-informed decisions and actions only if they are usable (ie, sufficiently easy and intuitive for users to navigate, interact with, and customize data visualizations) and useful in terms of being responsive to users’ information needs and generating valuable insights for guiding users’ understanding, reflection, decisions, and ultimately actions. Of the 2, usefulness appears to be most relevant to operationalizing actionability because usability is closely associated with a user’s technical and data analytical literacy and therefore may be considered a necessary but insufficient determinant of usefulness. However, our findings suggest that use and usability evaluations—whether via use of website analytics (22/89, 25%), experts’ evaluation (19/89, 21%), and user ratings (12/89, 14%)—are more common than evaluations of usefulness. Moreover, we found no evidence of systematic or rigorous evaluations of usefulness across the case studies of dashboards included in the scoping review. When an effort is made to assess usefulness, it is typically based on anecdotal user feedback (16/89, 18%), user engagement metrics derived from website analytics (14/89, 16%), or distal indicators such as citations or references to dashboards in academic publications (12/89, 14%). In this regard, we note that virtually none of the case studies of dashboards included in the review included an explicit theory of action that causally links dashboard use and usability to usefulness and impact of use, including the underlying mechanism that explains how use relates to outcomes (eg, drawing attention, facilitating learning and comprehension, persuading, and guiding choice among alternative actions).

Although user-centered design is frequently referenced in these case studies as the framework guiding the development of usable and useful dashboards, the development of these tools appears to be based mostly on dashboard developers’ expectations regarding how users *should* interact with, experience, and be influenced by using a dashboard, rather than on robust and thoughtful engagement with potential users and their expectations and needs. The fact that case studies that referenced using a co-design process to develop a dashboard were significantly fewer than case studies in which a dashboard was developed with no or minimal input from intended users (23/89, 26% compared to 39/89, 44% of all case studies, respectively), and that when collaborations were referenced, they most frequently involved scientific collaborations (45/89, 51%) and less frequently collaborations with users (15/89, 17%), appears to support this conclusion.

A third, less common conception of actionability that emerged from the scoping review (28/89, 32%) is focused on the degree of match between the insights that can be drawn from using a dashboard and the nature of the decision facing users. This conception of actionability is based on the recognition that the use of dashboards is often motivated by organizational goals and therefore ought to vary depending on whether strategic, tactical, or operational decisions are involved [21]. Thus, dashboards primarily designed for surveillance and monitoring (representing most of the case studies reviewed) can support operational decisions; dashboards that enable users to probe and analyze causes of health disparities or compare the efficacy of different intervention approaches can support tactical decisions; and dashboards that offer predictions (about 11/89, 12% of case studies of dashboards included in the review) or present data in context (eg, social determinants of health; about 27/89, 30% of case studies reviewed) can support strategic decisions regarding health policy and investments. This conception of actionability appears to be the least developed in the literature but may deserve greater attention from dashboard developers and researchers alike.

In summary, actionability assessment as applied to dashboards is more complex and multifaceted than portrayed in the literature on the topic. Among others, actionability is a function of user factors (capacity, needs, motivations, etc), characteristics of available data (quality, completeness, relevance, timeliness, granularity, etc), purpose (surveillance and monitoring, enlightenment, diagnosis, prediction and prognosis, prescription for action, etc), decisional goals (eg, strategic, tactical, or operational), desired impact (eg, on policy, practice, system change, and public education), and design elements (usability, functionality, interactivity, customization, adaptability, etc). It also requires consistent and informed use of dashboards and therefore is likely associated with the quality of dissemination efforts (ie, how users find out about the availability and value of using a dashboard); guidance regarding appropriate (and ethical) use; thoughtful integration with existing systems and users’ professional routines; and sustained sources of funding for technical support, maintenance, and continued improvement. Given this complexity, it is difficult to envision a standard set of metrics or indicators for studying and assessing actionability across applications and users of dashboards. A more productive path forward is to move away from a conception of actionability as a trait or property of usable and useful dashboards in favor of a more dynamic conception that understands actionability as a function of the iterative process used to conceive, design, deploy, evaluate, improve, and sustain dashboards that users find usable and useful given their goals, knowledge needs, and capacity.

## Discussion

### Principal Findings

Data dashboards can be a useful tool for improving knowledge translation, efficient and timely dissemination of insights from research, and equitable access for diverse users to critical health-related information. They can also support evidence-informed decision-making by serving multiple functions (eg, drawing attention and awareness to emerging challenges and monitoring change on existing ones, promoting more nuanced understanding of problems and potential solutions, facilitating goal setting, prioritizing, and sound allocation of resources) and can be valuable for data-focused collaborations. As public health data dashboards are poised to become more ubiquitous, it is imperative to proactively consider how they may be best designed to leverage public health data systems and meet the information needs of diverse audiences to support sound decisions regarding equitable and sustainable public health policies and practices [3,10]. However, as is evident from the findings of this scoping review, the scientific literature available to inform such efforts is considerably fragmented and lacking a standard, coherent focus regarding the goals, design, use, usefulness, and impact of these tools, as well as regarding factors (ie, conditions, circumstances, and support mechanisms) that explain variations in their use and usefulness across users and applications [3,8,10,11].

The rapid growth in public health data dashboard development in recent years—driven in part by the COVID-19 pandemic—may indicate that dashboard ecosystems are rapidly expanding along with technologies to support them, requiring conscientious approaches to dashboard design and applications, including improving on the adaptive or repurposing potential of these tools when public health priorities shift (as was the case for the COVID-19 dashboards). Despite this growth, our findings show that systematic and rigorously evaluated insights from the available literature regarding the optimal design, implementation, and improvement of public health dashboards are sparse and inconsistent, and therefore, insufficient to advance the future development and successful application of these tools at scale as well as support rigorous evaluations of their efficacy and public health impact.

Most of the case studies (36/89, 41%) included in the review were funded by the US government, with grants being the common funding mechanism used to support the development of public health dashboards. The dashboards considered in the literature were also more commonly hosted on university websites and designed via scientific collaborations. While our sample of case studies may be admittedly biased toward dashboards developed in academia, given that data were extracted from academic publications, legitimate questions nevertheless arise about the sustainability of such dashboards in light of familiar concerns about the long-term sustainability of these tools beyond classic project-based approaches to grant-funded work [120]. Still, as key drivers of innovation [121], including in public health [122], universities are uniquely positioned with expertise and institutional capacity to lead dashboard development efforts. This tension point may represent a fruitful area for further investigation and discussion.

Our review and synthesis also point to limited application of data dashboards in public health, including in relation to health inequities. The findings show that public health dashboards are primarily used for epidemiological surveillance and monitoring of various health risks. From a health equity perspective, such use of dashboards necessarily invites public health focus on deficits or disparities across subpopulations and communities; however, dashboards can be an equally effective tool for mapping and tracking assets (eg, available community resources that can be tapped in public health emergencies). Similarly, dashboards can have an important role in supporting effective public health advocacy by regularly monitoring the health policy-making and public opinion arenas, but these types of applications are significantly less common based on the findings of the review. In terms of intended users, the dashboards in our study were more commonly geared toward public health decision makers and policy makers than other public health stakeholders, such as the news media, public health advocates, and the general public. While the data used in dashboards are predominantly collected and shared by federal and state public health agencies, with institutional capacity for data management, curation, and interpretation [123], the case studies reviewed suggest that local data are increasingly available for integration into public health dashboards, but it is not clear whether the quality and representativeness of local data are sufficient for supporting sound decisions [124] or whether the design of dashboards for federal and state policy makers used is equally responsive to local decision makers’ knowledge needs and data use capacity. The finding that the practice of co-designing dashboards with users is rare, at least based on the cases reported in the literature on the topic, may raise concerns regarding the usability and usefulness of these tools to local public health decision makers.

### Limitations and Future Work

The scoping review methodology used in this study has several potential limitations. First, although we took multiple steps to ensure the rigor of our literature search and screening strategy, it is still possible that some relevant studies that met the study’s inclusion criteria were overlooked, including studies published after our search was concluded in mid-2023. However, by opting for a procedure designed to maximize recall (coverage) at the expense of precision (specificity), we were able to mitigate any potential bias due to omission of relevant studies. Second, and related to scope, the studies included in this scoping review were limited to public health data dashboards in the United States, whereas our search strategy identified a nontrivial number of relevant studies involving public health dashboards developed in other countries. Regions and countries around the world vary in terms of available public health data infrastructure, health systems, and public health conditions and priorities. Such international samples of case studies, while not directly comparable, may produce additional valuable insights and therefore deserve similar attention. Accordingly, we plan to conduct a separate, complementary scoping review of these additional case studies, using the same procedure and methodology implemented in this study, and compare the findings to the ones reported here, noting any similarities and differences between the 2 samples. Third, case studies of public health data dashboards that are available from the academic literature on this topic may overrepresent a particular type or subpopulation of dashboards (eg, dashboards developed and evaluated by university researchers) and therefore underrepresent the actual diversity of dashboard applications in public health, which may potentially bias our findings and conclusions. At the same time, our findings and conclusions are largely congruent with those reported by previous similar literature syntheses [2,4,12-14]. In addition, the next phase of our project, which involves coding and analysis of a probability sample of US federal and state public health data dashboards, will permit us to assess the degree and type of bias, if any, in the literature based on the findings of this scoping review. Finally, because the studies included in this scoping review vary considerably in the type and depth of the information provided, our data extraction and analysis, which focuses on detecting and synthesizing patterns of findings, may not be sufficiently robust to derive practical recommendations regarding the optimal design of actionable public health data dashboards; however, we believe this research contributes to advancing additional theory and research on this topic.

### Conclusions

Public health data dashboards have significant potential to support evidence-informed policy and practice decisions if they are actionable. The findings of the scoping review reveal a rather fragmented body of scholarship on this topic, which lacks a coherent and systematic focus on the various functions, design elements, causal mechanisms, conditions, and range of outcomes of dashboard use and their relationship with actionability across applications and diverse user groups. Notably absent from current scholarship are explicit theories of action that identify major factors (user-, design-, goal-, and context-related) that facilitate or impede informed use of these tools and explicate the mechanisms that link use with outcomes (eg, users’ knowledge, sensemaking, reflection, decisions, and actions) and ultimately impact practice or policy. Also notably missing are rigorously designed empirical studies that go beyond usability assessments to assess the usefulness of dashboards as a key dimension of actionability, as well as studies that tease out the relative advantages and disadvantages of different dashboard design philosophies and processes and produce practical recommendations. There is a significant opportunity for future research to advance both scholarship and practice regarding the design, deployment, and sustainability of actionable dashboards by addressing these existing gaps.

## Appendix

Multimedia Appendix 1 PRISMA-ScR checklist.

Multimedia Appendix 2 List and characteristics of case studies included in the scoping review.

## Abbreviations

CDC: Centers for Disease Control and Prevention
MeSH: Medical Subject Headings
PRISMA: Preferred Reporting Items for Systematic Reviews and Meta-Analyses
PRISMA-ScR: Preferred Reporting Items for Systematic reviews and Meta-Analyses extension for Scoping Reviews

## Data Availability

The datasets generated or analyzed during this study are available from the corresponding author on reasonable request.
