# Supplementary material for: Design, Application, and Actionability of US Public Health Data Dashboards: Scoping Review
Source: J Med Internet Res. 2025 May 21;27:e65283. doi: 10.2196/65283 (PMC12138306; doi:10.2196/65283)
Supplement: Multimedia Appendix 2 [file jmir_v27i1e65283_app2.docx]

**Multimedia Appendix 2: List and characteristics of case studies included in the scoping review**

| **Author and Year** | **Active**  **Dashboard** | **Access** | **Data source** | **Public Health Issue** | **Data Visualizations** |
| --- | --- | --- | --- | --- | --- |
| Anderson et al. 2019 | No | Open | State agencies | Opioid overdose deaths | Maps; Graphs / charts. |
| Backonja et al. 2022 | Yes | Open | Federal agencies; State agencies; Research organizations; Polling organizations. | Diabetes; Opioids; Physical activity; Nutrition; Smoking; Mental Health; Suicide; Injury; Violence; Oral Health | Graphs / charts; Timeline / trend. |
| Baxter et al. 2022 | N/A | Restricted | Federal agencies; State agencies; Research organizations; Health care facilities administrative data | COVID-19 | Graphs / charts; Tables; Timeline / trend. |
| Bilal et al. 2022 | Yes | Open | Federal agencies; Local agencies; Research organizations. | COVID-19 | Maps; Graphs / charts; Timeline / trend. |
| Bonham-Werling et al. 2021 | Yes | Open | Federal agencies; Health care facilities administrative data | COVID-19 | Maps; Graphs / charts. |
| Bors et al. 2015 | No | Restricted | Dashboard users | Childhood obesity | N/A |
| Brakefield et al. 2022 | N/A | Restricted | Federal agencies | Obesity | Graphs / charts |
| Brinkley et al. 2016 | Yes | Open | Research organizations | Craniofacial disorders | Graphs / charts; Matrix. |
| Chande et al. 2020 | Yes | Open | Federal agencies; State agencies; Media. | COVID-19 | Maps; Graphs / charts. |
| Claborn et al. 2022 | N/A | Restricted | Dashboard users | Drug overdose | N/A |
| Cocoros et al. 2021 | Yes | Restricted | Health care facilities administrative data | Monitor major diseases and conditions of interest on an in-care population | Maps; Graphs / charts. |
| Coelho et al. 2022 | Yes | Restricted | Research organizations; Media; International organizations. | COVID-19 | Maps; Graphs / charts; Timeline / trend; Heatmaps. |
| Cramer et al. 2022 | Yes | Open | Federal agencies; Research organizations; Media. | COVID-19 | Graphs / charts; Timeline / trend. |
| Curriero et al. 2021 | Yes | Open | Federal agencies; Google Trends; Canada provincial public health organizations | Lyme and tickborne diseases | Maps; Graphs / charts; Tables; Timeline / trend. |
| D'Agostino et al. 2022 | Yes | Restricted | Federal agencies; Dashboard users. | COVID-19 | Graphs / charts; Timeline / trend. |
| Dean et al. 2016 | Yes | Restricted | Patient/clinical data | Sleep and circadian disorders | Graphs / charts |
| Dixon et al. 2021a | Yes | Open | State agencies; Health care facilities administrative data; Patient/clinical data. | COVID-19 | Maps; Graphs / charts; Timeline / trend. |
| Dixon et al. 2021b | Yes | Open | State agencies | COVID-19 | Maps; Graphs / charts; Tables; Timeline / trend. |
| Dong et al. 2020 | Yes | Open | Federal agencies; State agencies; Local agencies; Media; International organization (WHO). | COVID-19 | N/A |
| Dong et al. 2022 | Yes | Open | Federal agencies; Media. | COVID-19 | Maps; Timeline / trend. |
| Dupuis et al. 2018 | Yes | Open | Dashboard users | Genetics | Maps; Graphs / charts; Tables. |
| Florez & Singh 2020 | No | N/A | Federal agencies; International agency/organization (WHO). | COVID-19 | Maps; Graphs / charts; Timeline / trend. |
| Foldy et al. 2004 | N/A | Restricted | Health care facilities administrative data | Epidemic Syndromes | Graphs / charts; Timeline / trend. |
| Fosdick et al. 2022 | Yes | Open | Patient/clinical data | COVID-19 | Graphs / charts |
| Gardner 2022 | Yes | Open | Federal agencies; International agency/organization (WHO). | COVID-19 | Maps; Graphs / charts; Tables; Timeline / trend. |
| Gesteland et al. 2012 | N/A | N/A | Health care facilities administrative data | Infectious disease | Maps; Graphs / charts; Timeline / trend. |
| Ghosh et al. 2019 | Yes | Open | Research organizations | Bioinformatics | Graphs / charts; Heatmaps; Hierarchical clustering. |
| Gourevitch et al. 2019 | Yes | Open | Federal agencies; State agencies; Local agencies. | Health status, health determinants, and health equity. | Maps; Tables. |
| Graham et al. 2021 | Yes | Open | Federal agencies | Clinical trials sponsorship and monopolistic practices (pharmaceuticals). | Graphs / charts; Tables; Timeline / trend; Network map. |
| Hashmi et al. 2023 | N/A | N/A | Health care facilities administrative data | Care needs in aging population | Maps; Tables. |
| Harris et al. 2018 | Yes | Restricted | Media | Foodborne illness | Maps |
| Hedberg et al. 2019 | Yes | Open | State agencies; Health care facilities administrative data. | Opioid addiction | Timeline / trend |
| Hilton et al. 2011 | No | Open | Federal agencies | Road traffic injuries | Maps |
| Hswen et al. 2022 | Yes | Restricted | Smartphones; research organizations. | COVID-19 | Maps |
| Hutchinson-Colas et al. 2023 | Yes | Open | Federal agencies; State agencies | Maternal mortality | Maps; Graphs / charts. |
| Ising et al. 2023 | Yes | Open | Health care facilities administrative data | Emergency department mental health visits | Maps; Graphs / charts; Tables; Timeline / trend. |
| Ji et al. 2013 | No | N/A | Media; open-source medical ontology | COVID-19 epidemic-related anxieties | Maps; Graphs / charts; Timeline / trend. |
| Jo et al. 2022 | Yes | Open | Web pages | COVID-19 vaccine website accessibility barriers | Maps; Graphs / charts; Tables. |
| Joshi et al. 2017 | Yes | Open | State agencies; Local agencies; Health care facilities administrative data. | HIV/AIDS | Maps; Graphs / charts; Tables. |
| Kallenbach et al. 2022 | N/A | N/A | Federal agencies; Health care facilities administrative data; Patient/clinical data. | COVID-19 | Tables |
| Kaul et al. 2020 | N/A | Restricted | Federal agencies; State agencies. | COVID-19 deaths | Maps; Graphs / charts; Tables. |
| Kianersi et al 2021 | No | Open | Federal agencies | Cigarette and E-cigarette use | Maps; Timeline / trend. |
| Kostkova et al. 2017 | N/A | N/A | Media | Vaccine hesitancy | Graphs / charts; Tables. |
| Krause 2015 | N/A | Restricted | Federal agencies; State agencies; Research organizations. | Access to health care | Maps; Graphs / charts; Timeline / trend. |
| Laurent et al. 2020 | Yes | Open | Federal agencies; State agencies; Local agencies. | Housing conditions and health outcomes | Graphs / charts; Tables; Maps; Timeline / Trend. |
| Le et al. 2022 | Yes | Open | Federal agencies | Cardiovascular disease (CVD) | Maps; Graphs / charts; Tables; Timeline / trend; Line plots for timeline. |
| Lechner et al. 2022 | Yes | Open | State agencies | Newborn screening (NBS) quality | Maps; Graphs / charts; Tables; Timeline / trend. |
| Lee et al. 2020 | Yes | Open | Federal agencies; Patient/clinical data. | Sepsis risk-standardized mortality rate | Maps; Graphs / charts; Tables; Automatic year play tool. |
| Liu et al. 2020 | Yes | Open | Federal agencies; Media. | COVID-19 | Maps; Graphs / charts; Tables; Timeline / trend. |
| Marshall et al. 2017 | Yes | Open | State agencies | Drug overdose | Maps; Graphs / charts. |
| Mast et al. 2021 | Yes | Open | Insurance claims data | COVID-19 | Maps; Graphs / charts; Timeline / trend; Heatmap. |
| Mayfield et al. 2020 | Yes | Open | Insurance claims data; Dashboard users | Healthcare access | Graphs / charts; Tables |
| Mirhaji et al. 2004 | N/A | N/A | Local agencies; Health care facilities administrative data; Patient/clinical data; OTC sale data from grocery stores and pharmacies; school and work absenteeism data; environmental safety data. | Infectious disease outbreaks | Graphs / charts; Tables. |
| Naughton et al. 2023 | Yes | Open | Research organizations; Media; Dashboard users. | COVID-19 | Maps; Graphs / charts. |
| Ngai et al. 2022 | N/A | Restricted | State agencies; Health care facilities administrative data; Patient/clinical data. | COVID-19 | Maps; Graphs / charts; Tables; Timeline / trend. |
| Ninkov & Sedig 2019 | N/A | N/A | Websites | Anti-vaccination/polarization of vaccine debate | Maps; Graphs / charts; Word cloud. |
| Pace et al. 2022 | Yes | Open | State agencies; Local agencies. | Drinking water quality | Maps; Data layers. |
| Patel et al. 2021 | N/A | N/A | Electronic forms | COVID-19; Vaccine hesitancy | Maps; Graphs / charts. |
| Patrick et al. 2017 | N/A | N/A | Federal agencies | HIV/AIDS | Graphs / charts |
| Peddireddy et al. 2020 | No | Open | Federal agencies; Industry; Media; Non-profit organization. | COVID-19 | Maps; Graphs / charts; Tables; Timeline / trend. |
| Penaia et al. 2021 | Yes | Open | Federal agencies; State agencies. | COVID-19 | Maps; Timeline / trend. |
| Petroni et al. 2021 | Yes | Open | Federal agencies; State agencies. | Environmental hazards | Maps |
| Reid et al. 2022 | Yes | Open | Dashboard users | Human poison exposures | Maps; Graphs / charts. |
| Runnels et al. 2021 | N/A | Restricted | State agencies; Health care facilities administrative data; Patient/clinical data; Insurance claims data. | Behavioral Health (mental health disorders, substance use disorders) | N/A |
| Ryan et al. 2016 | Yes | Open | Federal agencies; State agencies; | Childhood Obesity | Graphs / charts |
| Shaheen et al. 2021 | N/A | N/A | Federal agencies; Health care facilities administrative data. | COVID-19 Vaccine administrations | Maps; Graphs / charts; Timeline / trend. |
| Shi, A. et al. 2022 | No | Open | Federal agencies; Research organizations; Media. | COVID-19 | Maps; Graphs / charts. |
| Shi, Q. et al. 2022 | N/A | Restricted | Federal agencies; State agencies; Health care facilities administrative data; Patient/clinical data. | COVID-19 | Maps; Graphs / charts; Timeline / trend. |
| Smith et al. 2022 | Yes | Open | Federal agencies; PubMed publications. | Immunology (immune signatures) | Tables |
| Sopan et al. 2012 | N/A | Open | Federal agencies; Insurance claims data. | Community health | Maps; Graphs / charts; Tables; Heatmap. |
| Stone et al. 2007 | No | Restricted | Federal agencies; Local agencies. | Health services use post hurricane Katrina and Rita | Maps |
| Stone et al. 2019 | N/A | N/A | Insurance claims data | Opioid overdose | Graphs / charts; Tables; Timeline / trend. |
| Sullivan et al. 2022 | Yes | Open | Federal agencies; State agencies; Local agencies. | HIV/AIDS | Maps; Graphs / charts; Tables; Timeline / trend. |
| Sullivan et al. 2020 | Yes | Open | Federal agencies; State agencies; Local agencies; Insurance claims data. | HIV/AIDS | Maps; Graphs / charts; infographics. |
| Suri et al. 2022 | No | Restricted | State agencies; Local agencies; Research organizations. | COVID-19 | Maps; Graphs / charts; Tables; Timeline / trend. |
| Thompson et al. 2021 | No | Open | Dashboard users | COVID-19 | Graphs / charts. |
| Tsuchida et al. 2021 | N/A | Restricted | Health care facilities administrative data; Patient/clinical data. | Emergency department patient care) | Graphs / charts; Tables. |
| Valdiserri & Sullivan 2018 | Yes | Open | Federal agencies; State agencies; Local agencies. | HIV/AIDS | Maps; Graphs / charts. |
| Wahi & Dukach 2019 | N/A | Restricted or N/A | Health care facilities administrative data; Patient/clinical data; Laboratory | Catheter-associated urinary tract infections | Graphs / charts |
| Williams et al. 2021 | Yes | Open | Federal agencies; Research organizations; peer-reviewed literature; international agencies. | Chemical exposures | Graphs / charts; Tables; Data matrix. |
| Wilson et al. 2021 | N/A | Restricted | Federal agencies; Local agencies; Industry. | COVID-19 | Maps |
| Wissel et al. 2020 | No | Open | Federal agencies; State agencies; Local agencies; Research organizations; Health care facilities administrative data; Patient/clinical data; Media. | COVID-19 | Graphs / charts; Tables. |
| Wong et al. 2022 | Yes | Open | Health care facilities administrative data. | Ventilator-associated events (VAE) | Graphs / charts; Heat maps. |
| Wong et al. 2020 | N/A | N/A | Health care facilities administrative data; Insurance claims data. | Diabetes | Graphs / charts; Tables. |
| Wu et al. 2020 | N/A | Restricted | State agencies | Opioid overdose | Graphs / charts; Timeline / trend. |
| Yang et al. 2016 | No | Open | Media | Health information diffusion | Maps; Graphs / charts; Tables; Timeline / trend; Word cloud. |
| Yu et al. 2017 | N/A | Restricted | Federal agencies; Health care facilities administrative data; Patient/clinical data; Insurance claims data. | Access to patient care | Maps; Heatmaps. |
| Zheng et al. 2021 | Yes | Restricted | Health care facilities administrative data. | Health care-associated infections (HAIs) | Maps; Graphs / charts; Tables; Timeline / trend. |
| Zhu et al. 2021 | Yes | Open | Federal agencies; Research organizations; Media; WHO (International organization). | COVID-19 misinformation | Maps; Graphs / charts; Timeline / trend. |

# References:

Anderson J, Demeter N, Pasquires M y sol, Wirtz S. Using the CA Opioid Overdose Surveillance Dashboard to track opioid overdose deaths. *Online Journal of Public Health Informatics*. 2019;11(1). doi:[10.5210/ojphi.v11i1.9938](https://doi.org/10.5210/ojphi.v11i1.9938)

Backonja U, Park S, Kurre A, et al. Supporting rural public health practice to address local-level social determinants of health across Northwest states: Development of an interactive  visualization dashboard. *Journal of Biomedical Informatics*. 2022;129:N.PAG-N.PAG. doi:[10.1016/j.jbi.2022.104051](https://doi.org/10.1016/j.jbi.2022.104051)

Baxter L, Baynes J, Weaver A, et al. Development of the United States Environmental Protection Agency’s Facilities Status Dashboard for the COVID-19 Pandemic: Approach and Challenges. *International journal of public health*. 2022;67:1604761. doi:[10.3389/ijph.2022.1604761](https://doi.org/10.3389/ijph.2022.1604761)

Bilal U, McCulley E, Li R, et al. Tracking COVID-19 Inequities Across Jurisdictions Represented in the Big Cities Health Coalition (BCHC): The COVID-19 Health Inequities in BCHC Cities Dashboard. *American Journal of Public Health*. 2022;112(6):904-912. doi:[10.2105/ajph.2021.306708](https://doi.org/10.2105/ajph.2021.306708)

Bonham-Werling J, DeLonay AJ, Stephenson K, et al. Using Statewide Electronic Health Record and Influenza Vaccination Data to Plan and Prioritize COVID-19 Vaccine Outreach and Communications in Wisconsin  Communities. *American Journal of Public Health*. 2021;111(12):2111-2114. doi:[10.2105/ajph.2021.306524](https://doi.org/10.2105/ajph.2021.306524)

Bors PA, Kemner A, Fulton J, Stachecki J, Brennan LK. HKHC Community Dashboard: design, development, and function of a Web-based performance monitoring system. *Journal of public health management and practice : JPHMP*. 2015;21 Suppl 3:S36-S44. doi:[10.1097/PHH.0000000000000207](https://doi.org/10.1097/PHH.0000000000000207)

Brakefield WS, Ammar N, Shaban-Nejad A. An Urban Population Health Observatory for Disease Causal Pathway Analysis and Decision Support: Underlying Explainable Artificial Intelligence Model. *JMIR formative research*. 2022;6(7):e36055. doi:[10.2196/36055](https://doi.org/10.2196/36055)

Brinkley JF, Fisher S, Harris MP, et al. The FaceBase Consortium: a comprehensive resource for craniofacial researchers. *Development (Cambridge, England)*. 2016;143(14):2677-2688. doi:[10.1242/dev.135434](https://doi.org/10.1242/dev.135434)

Chande A, Lee S, Harris M, Hilley T, Andris C, Weitz JS. Real-time, interactive website for US-county level Covid-19 event risk assessment. Published online August 29, 2020:2020.08.24.20181271. doi:[10.1101/2020.08.24.20181271](https://doi.org/10.1101/2020.08.24.20181271)

Claborn K, Creech S, Conway FN, et al. Development of a digital platform to improve community response to overdose and prevention among harm reduction organizations. *Harm reduction journal*. 2022;19(1):62. doi:[10.1186/s12954-022-00636-2](https://doi.org/10.1186/s12954-022-00636-2)

Cocoros NM, Kirby C, Zambarano B, et al. RiskScape: A Data Visualization and Aggregation Platform for Public Health Surveillance Using Routine Electronic Health Record Data. *American Journal of Public Health*. 2021;111(2):269-276. doi:[10.2105/AJPH.2020.305963](https://doi.org/10.2105/AJPH.2020.305963)

Coelho D, Gupta N, Papenhausen E, Mueller K, IEEE. Patterns of Social Vulnerability - An Interactive Dashboard to Explore Risks to Public Health on the US County Level. In: *State University of New York (SUNY) System*. ; 2022:1-9. doi:[10.1109/VAHC57815.2022.10108527](https://doi.org/10.1109/VAHC57815.2022.10108527)

Cramer E, Huang Y, Wang Y, et al. The United States COVID-19 Forecast Hub dataset. *SCIENTIFIC DATA*. 2022;9(1). doi:[10.1038/s41597-022-01517-w](https://doi.org/10.1038/s41597-022-01517-w)

Curriero FC, Wychgram C, Rebman AW, et al. The Lyme and Tickborne Disease Dashboard: A map-based resource to promote public health awareness and research collaboration. *PLoS One*. 2021;16(12):e0260122. doi:[10.1371/journal.pone.0260122](https://doi.org/10.1371/journal.pone.0260122)

D’Agostino EM, Feger BJ, Pinzon MF, Bailey R, Kibbe WA. Democratizing Research With Data Dashboards: Data Visualization and Support to Promote Community Partner Engagement. *American Journal of Public Health*. 2022;112(S9):S850-S853. doi:[10.2105/ajph.2022.307103](https://doi.org/10.2105/ajph.2022.307103)

Dean DA 2nd, Goldberger AL, Mueller R, et al. Scaling Up Scientific Discovery in Sleep Medicine: The National Sleep Research Resource. *Sleep*. 2016;39(5):1151-1164. doi:[10.5665/sleep.5774](https://doi.org/10.5665/sleep.5774)

Dixon B, Grannis S, Tachinardi U, et al. Daily Visualization of Statewide COVID-19 Healthcare Data. In: *Indiana University System*. ; 2021:1-3. doi:[10.1109/VAHC53729.2020.00007](https://doi.org/10.1109/VAHC53729.2020.00007)

Dixon BE, Grannis SJ, McAndrews C, et al. Leveraging data visualization and a statewide health information exchange to support COVID-19 surveillance and response: Application of public health  informatics. *Journal of the American Medical Informatics Association : JAMIA*. 2021;28(7):1363-1373. doi:[10.1093/jamia/ocab004](https://doi.org/10.1093/jamia/ocab004)

Dong E, Du H, Gardner L. An interactive web-based dashboard to track COVID-19 in real time. *The Lancet Infectious Diseases*. 2020;20(5):533-534. doi:[10.1016/S1473-3099(20)30120-1](https://doi.org/10.1016/S1473-3099(20)30120-1)

Dong E, Ratcliff J, Goyea TD, et al. The Johns Hopkins University Center for Systems Science and Engineering COVID-19 Dashboard: data collection process, challenges faced, and lessons learned. *The Lancet Infectious diseases*. 2022;22(12):e370-e376. doi:[10.1016/S1473-3099(22)00434-0](https://doi.org/10.1016/S1473-3099(22)00434-0)

Dupuis JR, Bremer FT, Jombart T, Sim SB, Geib SM. mvmapper: Interactive spatial mapping of genetic structures. *Molecular ecology resources*. 2018;18(2):362-367. doi:[10.1111/1755-0998.12724](https://doi.org/10.1111/1755-0998.12724)

Florez H, Singh S. Online dashboard and data analysis approach for assessing COVID-19

case and death data. Published online June 8, 2020. doi:[10.12688/f1000research.24164.1](https://doi.org/10.12688/f1000research.24164.1)

Foldy SL, Biedrzycki PA, Baker BK, et al. The Public Health Dashboard: a surveillance model for bioterrorism preparedness. *Journal of public health management and practice : JPHMP*. 2004;10(3):234-240. doi:[10.1097/00124784-200405000-00007](https://doi.org/10.1097/00124784-200405000-00007)

Fosdick BK, Bayham J, Dilliott J, Ebel GD, Ehrhart N. Model-based evaluation of policy impacts and the continued COVID-19 risk at long term care facilities. *Infectious Disease Modelling*. 2022;7(3):463-472. doi:[10.1016/j.idm.2022.07.003](https://doi.org/10.1016/j.idm.2022.07.003)

Gardner L. The COVID-19 Dashboard for Real-time Tracking of the Pandemic: The Lasker-Bloomberg Public Service Award. *JAMA*. 2022;328(13):1295-1296. doi:[10.1001/jama.2022.15590](https://doi.org/10.1001/jama.2022.15590)

Gesteland PH, Livnat Y, Galli N, et al. The EpiCanvas infectious disease weather map: an interactive visual exploration of temporal and spatial correlations. *Journal of the American Medical Informatics Association : JAMIA*. 2012;19(6):954-959. doi:[10.1136/amiajnl-2011-000486](https://doi.org/10.1136/amiajnl-2011-000486)

Ghosh S, Datta A, Tan K, Choi H. SLIDE - a web-based tool for interactive visualization of large-scale - omics data. *Bioinformatics (Oxford, England)*. 2019;35(2):346-348. doi:[10.1093/bioinformatics/bty534](https://doi.org/10.1093/bioinformatics/bty534)

Gourevitch MN, Athens JK, Levine SE, Kleiman N, Thorpe LE. City-Level Measures of Health, Health Determinants, and Equity to Foster Population Health Improvement: The City Health Dashboard. *American Journal of Public Health*. 2019;109(4):585-592. doi:[10.2105/AJPH.2018.304903](https://doi.org/10.2105/AJPH.2018.304903)

Graham SS, Majdik ZP, Barbour JB, Rousseau JF. A dashboard for exploring clinical trials sponsorship and potential virtual monopolies. *JAMIA Open*. 2021;4(4):ooab089. doi:[10.1093/jamiaopen/ooab089](https://doi.org/10.1093/jamiaopen/ooab089)

Harris JK, Hinyard L, Beatty K, et al. Evaluating the Implementation of a Twitter-Based Foodborne Illness Reporting Tool in the City of St. Louis Department of Health. *Int J Environ Res Public Health*. 2018;15(5):833. doi:[10.3390/ijerph15050833](https://doi.org/10.3390/ijerph15050833)

Hashmi AZ, Christy J, Saxena S, Factora R. An age‐friendly population health dashboard geolocating by clinical and social determinant needs. *Health Services Research*. 2023;58 Suppl 1(Suppl 1):44-50. doi:[10.1111/1475-6773.14070](https://doi.org/10.1111/1475-6773.14070)

Hedberg K, Bui LT, Livingston C, Shields LM, Van Otterloo J. Integrating Public Health and Health Care Strategies to Address the Opioid Epidemic: The Oregon Health Authority’s Opioid Initiative. *Journal of public health management and practice : JPHMP*. 2019;25(3):214-220. doi:[10.1097/PHH.0000000000000849](https://doi.org/10.1097/PHH.0000000000000849)

Hilton B, Horan T, Burkhard R, Schooley B. SafeRoadMaps: Communication of location and density of traffic fatalities through spatial visualization and heat map analysis. *INFORMATION VISUALIZATION*. 2011;10(1):82-96. doi:[10.1057/ivs.2010.14](https://doi.org/10.1057/ivs.2010.14)

Hswen Y, Yom-Tov E, Murti V, et al. Covidseeker: A Geospatial Temporal Surveillance Tool. *International journal of environmental research and public health*. 2022;19(3). doi:[10.3390/ijerph19031410](https://doi.org/10.3390/ijerph19031410)

Hutchinson-Colas JA, Balica A, Chervenak FA, et al. New Jersey maternal mortality dashboard: an interactive social-determinants-of-health tool. *Journal of Perinatal Medicine*. 2023;51(2):188-196. doi:[10.1515/jpm-2021-0673](https://doi.org/10.1515/jpm-2021-0673)

Ising A, Waller A, Frerichs L. Evaluation of an Emergency Department Visit Data Mental Health Dashboard. *Journal of public health management and practice : JPHMP*. 2023;29(3):369-376. doi:[10.1097/PHH.0000000000001727](https://doi.org/10.1097/PHH.0000000000001727)

Ji X, Chun S, Geller J, IEEE. Monitoring Public Health Concerns Using Twitter Sentiment Classifications. In: *New Jersey Institute of Technology*. ; 2013:335-344. doi:[10.1109/ICHI.2013.47](https://doi.org/10.1109/ICHI.2013.47)

Jo G, Habib D, Varadaraj V, et al. COVID-19 vaccine website accessibility dashboard. *Disability and health journal*. 2022;15(3):101325. doi:[10.1016/j.dhjo.2022.101325](https://doi.org/10.1016/j.dhjo.2022.101325)

Joshi A, Amadi C, Katz B, Kulkarni S, Nash D. A Human-Centered Platform for HIV Infection Reduction in New York: Development and Usage Analysis of the Ending the Epidemic (ETE) Dashboard. *JMIR public health and surveillance*. 2017;3(4):e95. doi:[10.2196/publichealth.8312](https://doi.org/10.2196/publichealth.8312)

Kallenbach L, Whipple S, Bonafede M. EPH60 Real-World Data Dashboard Integrating Public Health & EHR Data for Identifying COVID-19 Vaccination Gaps in the US. *Value in Health*. 2022;25(7):S445. doi:[10.1016/j.jval.2022.04.813](https://doi.org/10.1016/j.jval.2022.04.813)

Kaul S, Coleman C, Gotz D. A rapidly deployed, interactive, online visualization system to support fatality management during the coronavirus disease 2019 (COVID-19) pandemic. *Journal of the American Medical Informatics Association : JAMIA*. 2020;27(12):1943-1948. doi:[10.1093/jamia/ocaa146](https://doi.org/10.1093/jamia/ocaa146)

Kianersi S, Zhang Y, Rosenberg M, Macy JT. Prevalence of e-cigarette use (2016 to 2018) and cigarette smoking (2012 to 2019) among U.S. adults by state: An interactive data visualization dashboard. *Drug and alcohol dependence*. 2021;218:108361. doi:[10.1016/j.drugalcdep.2020.108361](https://doi.org/10.1016/j.drugalcdep.2020.108361)

Kostkova P, Mano V, Larson H, Schulz W, ACM. Who is Spreading Rumours about Vaccines? Influential User Impact Modelling in Social Networks. In: *University of London*. ; 2017:48-52. doi:[10.1145/3079452.3079505](https://doi.org/10.1145/3079452.3079505)

Krause DD. Data Lakes and Data Visualization: An Innovative Approach to Address the Challenges of Access to Health Care in Mississippi. *Online journal of public health informatics*. 2015;7(3):e225. doi:[10.5210/ojphi.v7i3.6047](https://doi.org/10.5210/ojphi.v7i3.6047)

Laurent AA, Matheson A, Escudero K, Lazaga A. Linking Health and Housing Data to Create a Sustainable Cross-Sector Partnership. *American Journal of Public Health*. 2020;110(S2):S222-S224. doi:[10.2105/AJPH.2020.305693](https://doi.org/10.2105/AJPH.2020.305693)

Le P, Casper M, Vaughan AS. A Dynamic Visualization Tool of Local Trends in Heart Disease and Stroke Mortality in the United States. *Preventing chronic disease*. 2022;19:E57. doi:[10.5888/pcd19.220076](https://doi.org/10.5888/pcd19.220076)

Lechner C, Rumpler M, Dorley MC, Li Y, Ingram A, Fryman H. Developing an Online Dashboard to Visualize Performance Data-Tennessee Newborn Screening Experience. *International journal of neonatal screening*. 2022;8(3). doi:[10.3390/ijns8030049](https://doi.org/10.3390/ijns8030049)

Lee MT, Lin FC, Chen ST, et al. Web-Based Dashboard for the Interactive Visualization and Analysis of National Risk-Standardized Mortality Rates of Sepsis in the US. *Journal of medical systems*. 2020;44(2):54. doi:[10.1007/s10916-019-1509-9](https://doi.org/10.1007/s10916-019-1509-9)

Liu S, Wall E, Patel SA, Park Y. COVID-19 Health Equity Dashboard - Addressing Vulnerable Populations. Published online August 24, 2020. doi:[10.31219/osf.io/2frha](https://doi.org/10.31219/osf.io/2frha)

Marshall BDL, Yedinak JL, Goyer J, Green TC, Koziol JA, Alexander-Scott N. Development of a Statewide, Publicly Accessible Drug Overdose Surveillance and Information System. *American journal of public health*. 2017;107(11):1760-1763. doi:[10.2105/AJPH.2017.304007](https://doi.org/10.2105/AJPH.2017.304007)

Mast TC, Heyman D, Dasbach E, Roberts C, Goveia MG, Finelli L. Planning for monitoring the introduction and effectiveness of new vaccines using real-word data and geospatial visualization: An example using rotavirus vaccines  with potential application to SARS-CoV-2. *Vaccine X*. 2021;7:100084. doi:[10.1016/j.jvacx.2021.100084](https://doi.org/10.1016/j.jvacx.2021.100084)

Mayfield CA, Gigler ME, Snapper L, et al. Using cloud-based, open-source technology to evaluate, improve, and rapidly disseminate community-based intervention data. *Journal of the American Medical Informatics Association : JAMIA*. 2020;27(11):1741-1746. doi:[10.1093/jamia/ocaa181](https://doi.org/10.1093/jamia/ocaa181)

Mirhaji P, Richesson R, Turley J, Zhang J, Smith J. Public health situation awareness, towards a semantic approach. In: Dasarathy B, ed. *University of Texas System*. Vol 5434. ; 2004:339-350. doi:[10.1117/12.541189](https://doi.org/10.1117/12.541189)

Naughton CC, Roman FAJ, Alvarado AGF, et al. Show us the data: global COVID-19 wastewater monitoring efforts, equity, and gaps. *FEMS Microbes*. 2023;4:xtad003. doi:[10.1093/femsmc/xtad003](https://doi.org/10.1093/femsmc/xtad003)

Ngai S, Sell J, Baig S, et al. Built by epidemiologists for epidemiologists: an internal COVID-19 dashboard for real-time situational awareness in New York City. *JAMIA Open*. 2022;5(2):ooac029. doi:[10.1093/jamiaopen/ooac029](https://doi.org/10.1093/jamiaopen/ooac029)

Ninkov A, Sedig K. VINCENT: A visual analytics system for investigating the online vaccine debate. *Online journal of public health informatics*. 2019;11(2):e5. doi:[10.5210/ojphi.v11i2.10114](https://doi.org/10.5210/ojphi.v11i2.10114)

Pace C, Fencl A, Baehner L, Lukacs H, Cushing LJ, Morello-Frosch R. The Drinking Water Tool: A Community-Driven Data Visualization Tool for Policy Implementation. *International journal of environmental research and public health*. 2022;19(3). doi:[10.3390/ijerph19031419](https://doi.org/10.3390/ijerph19031419)

Patel J, Dzomba B, Vo H, Von Nessen-Scanlin S, Siminoff L, Wu H. A Health IT-Empowered Integrated Platform for Secure Vaccine Data Management and Intelligent Visual Analytics and Reporting. In: Bier N, Fred A, Gamboa H, eds. *Pennsylvania Commonwealth System of Higher Education (PCSHE)*. ; 2021:522-531. doi:[10.5220/0010843700003123](https://doi.org/10.5220/0010843700003123)

Patrick R, Greenberg A, Magnus M, Opoku J, Kharfen M, Kuo I. Development of an HIV Testing Dashboard to Complement the HIV Care Continuum Among MSM, PWID, and Heterosexuals in Washington, DC, 2007-2015. *Journal of acquired immune deficiency syndromes (1999)*. 2017;75 Suppl 3(Suppl 3):S397-S407. doi:[10.1097/QAI.0000000000001417](https://doi.org/10.1097/QAI.0000000000001417)

Peddireddy A, Xie D, Patil P, et al. From 5Vs to 6Cs: Operationalizing Epidemic Data Management with COVID-19 Surveillance. In: Wu X, Jermaine C, Xiong L, et al., eds. *University of Virginia*. ; 2020:1380-1387. doi:[10.1109/BigData50022.2020.9378435](https://doi.org/10.1109/BigData50022.2020.9378435)

Penaia CS, Morey BN, Thomas KB, et al. Disparities in Native Hawaiian and Pacific Islander COVID-19 Mortality: A Community-Driven Data Response. *American Journal of Public Health*. 2021;111(S2):S49-S52. doi:[10.2105/AJPH.2021.306370](https://doi.org/10.2105/AJPH.2021.306370)

Petroni M, Howard S, Howell I, Collins M. NYenviroScreen: An open-source data driven method for identifying potential environmental justice communities in New York State. *ENVIRONMENTAL SCIENCE & POLICY*. 2021;124:348-358. doi:[10.1016/j.envsci.2021.07.004](https://doi.org/10.1016/j.envsci.2021.07.004)

Reid NE, Johnson-Arbor K, Smolinske S, Litovitz T. 2020 webPOISONCONTROL data summary. *The American journal of emergency medicine*. 2022;54:184-195. doi:[10.1016/j.ajem.2022.02.014](https://doi.org/10.1016/j.ajem.2022.02.014)

Runnels P, Coran JJ, Goldman ML, Pronovost P. Utilizing a Dashboard to Promote System-Wide Value in Behavioral Health. *Population health management*. 2021;24(4):427-429. doi:[10.1089/pop.2020.0233](https://doi.org/10.1089/pop.2020.0233)

Ryan K, Pillai P, Remington PL, Malecki K, Lindberg S. Development of an Obesity Prevention Dashboard for Wisconsin. *WMJ : official publication of the State Medical Society of Wisconsin*. 2016;115(5):224-227.

Shaheen AW, Ciesco E, Johnson K, Kuhnen G, Paolini C, Gartner G. Interactive, on-line visualization tools to measure and drive equity in COVID-19 vaccine administrations. *Journal of the American Medical Informatics Association : JAMIA*. 2021;28(11):2451-2455. doi:[10.1093/jamia/ocab180](https://doi.org/10.1093/jamia/ocab180)

Shi A, Gaynor SM, Dey R, Zhang H, Quick C, Lin X. COVID-19 Spread Mapper: a multi-resolution, unified framework and open-source tool. *Bioinformatics (Oxford, England)*. 2022;38(9):2661-2663. doi:[10.1093/bioinformatics/btac129](https://doi.org/10.1093/bioinformatics/btac129)

Shi Q, Herbert C, Ward DV, et al. COVID-19 Variant Surveillance and Social Determinants in Central Massachusetts: Development Study. *JMIR formative research*. 2022;6(6):e37858. doi:[10.2196/37858](https://doi.org/10.2196/37858)

Smith KC, Chawla DG, Dhillon BK, et al. A  curated collection of human vaccination response signatures. *Scientific data*. 2022;9(1):678. doi:[10.1038/s41597-022-01558-1](https://doi.org/10.1038/s41597-022-01558-1)

Sopan A, Noh A, Karol S, Rosenfeld P, Lee G, Shneiderman B. Community Health Map: A geospatial. and multivariate data visualization tool for public health datasets. *GOVERNMENT INFORMATION QUARTERLY*. 2012;29(2):223-234. doi:[10.1016/j.giq.2011.10.002](https://doi.org/10.1016/j.giq.2011.10.002)

Stone AB, Jones MR, Rao N, Urman RD. A Dashboard for Monitoring Opioid-Related Adverse Drug Events Following Surgery Using a National Administrative Database. *Am J Med Qual*. 2019;34(1):45-52. doi:[10.1177/1062860618782646](https://doi.org/10.1177/1062860618782646)

Stone G, Lekht A, Burris N, Williams C. Data collection and communications in the public health response to a disaster: rapid population estimate surveys and the Daily Dashboard in post-Katrina New  Orleans. *Journal of public health management and practice : JPHMP*. 2007;13(5):453-460. doi:[10.1097/01.phh.0000285196.16308.7d](https://doi.org/10.1097/01.phh.0000285196.16308.7d)

Sullivan PS, Woodyatt C, Koski C, et al. A Data Visualization and Dissemination Resource to Support HIV Prevention and Care at the Local Level: Analysis and Uses of the AIDSVu Public Data Resource. *Journal of Medical Internet Research*. 2020;22(10):N.PAG-N.PAG. doi:[10.2196/23173](https://doi.org/10.2196/23173)

Sullivan PS, Woodyatt CR, Kouzouian O, et al. America’s HIV Epidemic Analysis Dashboard: Protocol for a Data Resource to Support Ending the HIV Epidemic in the United States. *JMIR public health and surveillance*. 2022;8(2):e33522. doi:[10.2196/33522](https://doi.org/10.2196/33522)

Suri A, Askari M, Calder J, Branas C, Rundle A. A real-time COVID-19 surveillance dashboard to support epidemic response in Connecticut: lessons from an academic-health department partnership. *Journal of the American Medical Informatics Association : JAMIA*. 2022;29(5):958-963. doi:[10.1093/jamia/ocac025](https://doi.org/10.1093/jamia/ocac025)

Thompson M, Belval E, Dilliott J, Bayham J. Supporting Wildfire Response During a Pandemic in the United States: the COVID-19 Incident Risk Assessment Tool. *FRONTIERS IN FORESTS AND GLOBAL CHANGE*. 2021;4. doi:[10.3389/ffgc.2021.655493](https://doi.org/10.3389/ffgc.2021.655493)

Tsuchida RE, Haggins AN, Perry M, et al. Developing an electronic health record–derived health equity dashboard to improve learner access to data and metrics. *AEM Education and Training*. 2021;5(S1):S116-S120. doi:[10.1002/aet2.10682](https://doi.org/10.1002/aet2.10682)

Valdiserri RO, Sullivan PS. Data Visualization Promotes Sound Public Health Practice: The AIDSvu Example. *AIDS education and prevention : official publication of the International Society for AIDS Education*. 2018;30(1):26-34. doi:[10.1521/aeap.2018.30.1.26](https://doi.org/10.1521/aeap.2018.30.1.26)

Wahi MM, Dukach N. Visualizing Infection Surveillance Data for Policymaking Using Open Source Dashboarding. *Applied clinical informatics*. 2019;10(3):534-542. doi:[10.1055/s-0039-1693649](https://doi.org/10.1055/s-0039-1693649)

Williams AJ, Lambert JC, Thayer K, Dorne JLCM. Sourcing data on chemical properties and hazard data from the US-EPA CompTox Chemicals Dashboard: A practical guide for human risk assessment. *Environment international*. 2021;154:106566. doi:[10.1016/j.envint.2021.106566](https://doi.org/10.1016/j.envint.2021.106566)

Wilson G, Ball M, Szczesny P, et al. Health Intelligence Atlas: A Core Tool for Public Health Intelligence. *APPLIED CLINICAL INFORMATICS*. 2021;12(04):944-953. doi:[10.1055/s-0041-1735973](https://doi.org/10.1055/s-0041-1735973)

Wissel BD, Van Camp PJ, Kouril M, et al. An interactive online dashboard for tracking COVID-19 in U.S. counties, cities, and states in real time. *Journal of the American Medical Informatics Association*. 2020;27(7):1121-1125. doi:[10.1093/jamia/ocaa071](https://doi.org/10.1093/jamia/ocaa071)

Wong AKI, Kim H, Charpignon ML, et al. A Method to Explore Variations of Ventilator-Associated Event Surveillance Definitions in Large Critical Care Databases in the United States. *Critical care explorations*. 2022;4(11):e0790. doi:[10.1097/CCE.0000000000000790](https://doi.org/10.1097/CCE.0000000000000790)

Wong T, Brovman EY, Rao N, Tsai MH, Urman RD. A Dashboard Prototype for Tracking the Impact of Diabetes on Hospital Readmissions Using a National Administrative Database. *J Clin Med Res*. 2020;12(1):18-25. doi:[10.14740/jocmr.v12i1.4029](https://doi.org/10.14740/jocmr.v12i1.4029)

Wu E, Villani J, Davis A, et al. Community dashboards to support data-informed decision-making in the HEALing communities study. *Drug and alcohol dependence*. 2020;215:108331-108331. doi:[10.1016/j.drugalcdep.2020.108331](https://doi.org/10.1016/j.drugalcdep.2020.108331)

Yang JA, Tsou MH, Jung CT, et al. Social media analytics and research testbed (SMART): Exploring spatiotemporal          patterns of human dynamics with geo-targeted social media messages. *Big Data & Society*. 2016;3(1):2053951716652914. doi:[10.1177/2053951716652914](https://doi.org/10.1177/2053951716652914)

Yu Z, Pepe K, Rust G, Ramirez-Marquez JE, Zhang S, Bonnet B. Patient-provider geographic map: An interactive visualization tool of patients’ selection of health care providers. In: *2017 IEEE Workshop on Visual Analytics in Healthcare (VAHC)*. ; 2017:1-8. doi:[10.1109/VAHC.2017.8387494](https://doi.org/10.1109/VAHC.2017.8387494)

Zheng S, Edwards JR, Dudeck MA, et al. Building an Interactive Geospatial Visualization Application for National Health Care-Associated Infection Surveillance: Development Study. *JMIR public health and surveillance*. 2021;7(7):e23528. doi:[10.2196/23528](https://doi.org/10.2196/23528)

Zhu Z, Meng K, Caraballo J, et al. A Dashboard for Mitigating the COVID-19 Misinfodemic. In: *Proceedings of the 16th Conference of the European Chapter of the Association for Computational Linguistics: System Demonstrations*. Association for Computational Linguistics; 2021:99-105. doi:[10.18653/v1/2021.eacl-demos.12](https://doi.org/10.18653/v1/2021.eacl-demos.12)
